# Supplementary material for: Differences in Antioxidant Potential of Allium cepa Husk of Red, Yellow, and White Varieties
Source: Antioxidants (Basel). 2022 Jun 24;11(7):1243. doi: 10.3390/antiox11071243 (PMC9311763; doi:10.3390/antiox11071243)
Supplement: Supplementary file 1 [file antioxidants-11-01243-s001.zip › antioxidants-1773793-supplementary.pdf]

Table S1. The mass parameters and identification characteristics of all manually selected compounds

Metabolite name	Adduct type	Ontology	Total score
3,4-Dihydroxybenzoic acid	[M-H]-	Hydroxybenzoic acid derivatives	92
Vanillic acid	[M-H]-	M-methoxybenzoic acids and derivatives	90
Gallic acid	[M-H]-	Gallic acids	88.5
Benzoic acid 2O, O-Hex; PlaSMA ID-666	[M-H]-	Phenolic glycosides	93.1
w/o MS2:Lecanoric Acid	[M-H]-	Depsides and depsidones	97.7
w/o MS2:Feruloyl quinic acid (isomer of 886, 887); PlaSMA ID-888	[M-H]-	Quinic acid and derivatives	96.1
Citric acid	[M-H]-	Tricarboxylic acids and derivatives	92.4
5-Ketogluconic acid	[M-H]-	Medium-chain hydroxy acids and derivatives	82
w/o MS2:Gluconic acid	[M-H]-	Medium-chain hydroxy acids and derivatives	99.8
w/o MS2:Lipoic acid	[M-H]-	Lipoic acids and derivatives	99.9
Bergapten	[M2H]-	5-methoxypsoralens	70.2
Dihydrochalcone	[M-H]-		73.3
w/o MS2:Oxazepam; LC-ESI-ITFT; MS2; CE	[M-H]-	1,4-benzodiazepines	73.9
w/o MS2:Lorazepam	[M-H]-	1,4-benzodiazepines	81
Tectorigenin	[M-H]-	Isoflavones	88.9
Quercetin	[M-H]-	Flavonols	91.6
w/o MS2:Taxifolin	[M-H]-	Flavanonols	85.3
Isoquercetrin	[M-H]-	Flavonoid-3-O-glycosides	94.3
Spiraeoside	[M-H]-	Flavonoid O-glycosides	90
3'-methoxy-4',5,7-trihydroxyflavonol	[M-H]-	Flavonols	85.7
w/o MS2:Myricetin	[M-H]-	Flavonols	98.7
Quercetin-3,4'-O-di-beta-glucoside	[M-H]-	Flavonoid-3-O-glycosides	84.9
Hyperoside; LC-ESI-QTOF; MS2; CE	[2M-H]-	Flavonoid-3-O-glycosides	81.8
w/o MS2:Laricitrin	[M-H]-	Flavonols	98.4
w/o MS2:5,7-dihydroxy-3,8-dimethoxy-2-phenyl-4H-chromen-4-one	[M-H]-	8-O-methylated flavonoids	93.3
w/o MS2:Haematommone	[M-H]-	Hydroxyanthraquinones	92.4
w/o MS2:skimmin	[M-H2O-H]-	Coumarin glycosides	93.3
hyperoside	[M-H]-		82.5
w/o MS2:3-O-acetylpadmatin	[M-H]-	7-O-methylated flavonoids	96.9
Isorhamnetin 3-galactoside	[M-H]-		87.7
